# Domain-Based Analysis of Colon Polyp in CT Colonography Using Image-Processing Techniques

**DOI:** 10.31557/APJCP.2019.20.2.629

**Published:** 2019

**Authors:** K N Manjunath, P C Siddalingaswamy, G K Prabhu

**Affiliations:** 1 *Department of Computer Science and Engineering, Manipal Institute of Technology, Manipal Academy of Higher Education, Manipal, *; 2 *Department of Electronics and Communication, Manipal University, Jaipur, India. *

**Keywords:** Computer, aided detection and diagnosis, segmentation, shape analysis, colon polyp, visualization

## Abstract

**Background:**

The purpose of the research was to improve the polyp detection accuracy in CT Colonography (CTC) through effective colon segmentation, removal of tagged fecal matter through Electronic Cleansing (EC), and measuring the smaller polyps.

**Methods:**

An improved method of boundary-based semi-automatic colon segmentation with the knowledge of colon distension, an adaptive multistep method for the virtual cleansing of segmented colon based on the knowledge of Hounsfield Units, and an automated method of smaller polyp measurement using skeletonization technique have been implemented.

**Results:**

The techniques were evaluated on 40 CTC dataset. The segmentation method was able to delineate the colon wall accurately. The submerged colonic structures were preserved without soft tissue erosion, pseudo enhanced voxels were corrected, and the air-contrast layer was removed without losing the adjacent tissues. The smaller polyp of size less than <10mm was detected correctly. The results were statistically validated qualitatively and quantitatively. Segmented colons were validated through volumetric overlap computation, and accuracy of 95.826±0.6854% was achieved. In polyp measurement, the paired t-test method was applied to compare the difference with ground truth and at α=5%, t=0.9937 and p=0.098 was achieved. The statistical values of TPR=90%, TNR=82.3% and accuracy=88.31% were achieved.

**Conclusion:**

An automated system of polyp measurement has been developed starting from colon segmentation to improve the existing CTC solutions. The analysis of domain-based approach of polyp has given good results. A prototype software, which can be used as a low-cost polyp diagnosis tool, has been developed.

## Introduction

**Figure 1:**
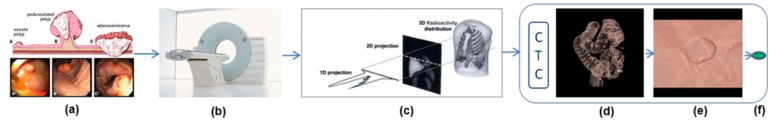
CT Colonography Workflow. a) Polyp growth [John Hopkins Medicine 2016], b) CT scanning [SIEMENS, 2017], c) 2D image reconstruction [Kalender 2012], d) Desired volume of interest extraction, e) Endoluminal view of colon, and f) Acceptance of result by radiologist

CT Colonography (CTC) is a non-invasive medical imaging-based technology for polyp diagnosis using Computer Aided Assessment system (CAA). The specific area of investigation is solving the technical problems in polyp diagnosis using CTC images. The workflow of CTC is shown in Figure. 1. The standard scanning protocol involves empty colon preparation (Figure. 1a), Multi-Detector CT (MDCT) image acquisition of the abdomen with least slice thickness (ST) (1b), reconstruction of 3D volume in *R*^3^ dimension from the 2D axial slices in *R*^2^ (1c), colon segmentation (1d), delineation of polyps through image processing techniques (1e) and visualization (1d, 1f) using 2D MPR (Multi Planar Reformatted) and 3D visualization techniques. The importance of polyp is decided by its size, shape, type, and grade of dysplasia out of which the first two are measurable in CTC and the other two through biopsy. The size is a maximum diameter measured (in mm) within the mass. Sizes are categorized as 1-5mm, 6-9 mm and greater than 10 mm (Sleisenger et al., 2010). A polyp of size more than 10 mm has more chances of becoming colon cancer. The 6-9mm range is also considered significant as it can show an advanced histology (Summers et al., 2009) and its diameter can exceed 10 mm over a period of time. 

The main technical limitations in CTC (problem) are inaccurate measurement and variations in true positive and true negative rates. For accurate measurement, an improved engineering solution is required for radiologists (clients), and they decide whether the research has solved the problem (scope). The technical details of colon segmentation, electronic cleansing (EC), and polyp measurements are discussed below in detail.

Segmenting the colon without sparing the base of the colonic structures is a desired step in polyp analysis. Methods discussed in colon segmentation were K-Means clustering (Terry, 2004), level set method (Franazek et al., 2006; Gross et al., 2009), active contours, template matching (Breier et al., 2011; Namias et al., 2016), Fuzzy C thresholding (Franazek et al., 2006), threshold-based region growing (Tian et al., 2001), volume thresholding (Yoshida et al., 2012; Cai et al., 2013) and confidence connected region growing technique (Lee et al., 2011). In the K-Means method, selection of different regions is difficult, and it entirely depends on voxel intensity distribution. The boundary was not properly delineated due to variation in the intensity levels. Level set and active contours perfectly hold good for outer boundary detection of soft tissues, but they fail to identify the base of the colonic structures. Fuzzy C thresholding works when the contrast of VOI is good, and it is difficult to define a valid threshold to differentiate colon from neighboring anatomies. The overlapping voxels of contrast agent in the colon and the bone density have lead to segmentation leak problems in case of region growing methods. This results in the wrong definition of the colon after segmentation. Volume thresholding purely depends on the threshold selection manually and it is not a preferred technique in the case of automated segmentation methods. The effective colon extraction at the colon wall needs to be improved because the base of the soft tissue structure is the key information to measure the polyp and width and height.

Even though the CTC protocol recommends for empty intestine before the CTC scan by using the suction pump, this procedure may cause discomfort in elderly people. To avoid this, the CTC scan is performed by retaining this oral contrast in the colon. Later, after image acquisition, this contrast is removed through image processing (EC) without tampering the anatomical details. Electronic Cleansing refers to the removal of tagged materials and the contrast agent from the colon using the image processing. The techniques discussed were morphological and convolution based processing (Zalis et al., 2004), voxel classification (Wang et al., 2005; Netto et al., 2017), arithmetic operation using vertical vector motion flow (Yamamoto et al., 2009), scale-invariant Gaussian derivatives (Serlie et al., 2010), local roughness analysis, mosaic decomposition, processing based on material composition (Serlie et al., 2010; Lee et al., 2014) and processing images based on dual energy CT (Cai et al., 2013). In Morphological and convolution based processing filtering techniques were discussed. Even though the results were good, the method was not a generalized solution as it was evaluated only on a limited number of the phantom dataset.

In Voxel classification, the regions with specific *HU* associated with colonic contents were processed. The results were promising, and they inferred that knowledge of *HU* of colonic contents can do better virtual cleansing without any artifact. In vertical vector motion flow technique, the uneven tagged areas were not cleansed properly. It was a major limitation of the study and even the normal structure was eroded to excessive filtering due to which the polyp details were lost. Scale invariant Gaussian derivatives involved the cleaning of colon by computing the Gaussian derivatives of tagged materials. The method was applied only on homogeneously tagged areas and it did not work for partially tagged areas. Dual energy CT is a better alternate solution for EC, but the image acquisition can lead the patient to more radiation exposure and processing such images is computationally expensive. Despite these methods, there exist a few post processing artifacts such as incomplete cleansing; soft tissue erosion and removal of air contrast boundary, which may lead to wrong measurement of polyp. An improved adaptive method which should work with images captured with various tube voltages (kVp, peak kilo voltage) is required.

Methods discussed in smaller polyp measurement were based on geometrical features such as mean, shape index and principal curvature, curvature-based region growing (Lee et al., 2011) and neural network based (Huang et al., 2010). In SVM, the authors conclude that limited shape information of the polyp is the major drawback to conclude anything about the size of the polyp. Smaller polyps (size < 5mm) were underestimated by CTC in a study and the sensitivity for measuring these is too low and there are differences of opinion about their clinical significance (Song et al., 2014). The complexity involved in measurement of a smaller polyp is due to its irregular geometrical shape (Lefere and Gryspeerdt, 2011; Weber et al., 2017). Pareto front approach uses Curvature-based region growing technique. The limitation in pareto front approach is the selection of right operating point. In many literatures, the properties of the polyp considered are, intensity distribution, curvedness of the structure, amount of deformation and protrusion. Due to clinical significance of smaller polyps and the current technical limitations, still, there is scope for improving the accuracy.

The goal of the study is to provide improved image processing methods for colon segmentation, virtual cleansing of fecal tagged CTC images and to measure smaller polyp from radiologist perspective of colon analysis. The methodology and the results about these objectives are discussed in separate sections.

## Materials and Methods

**Figure 2:**
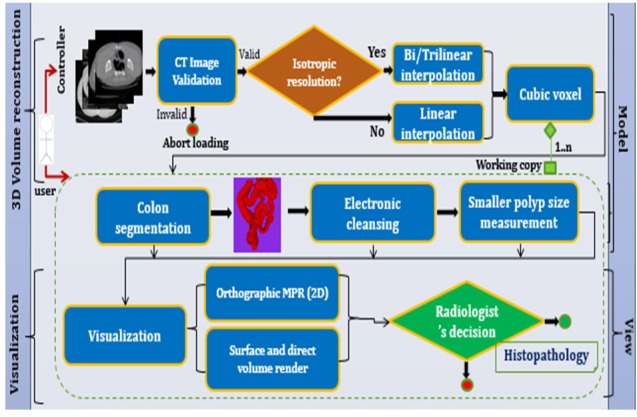
Block Diagram of the Proposed Methodology Using MVC Design Pattern of Software Engineering

Hybrid methods of image processing are gaining popularity due to less accuracy from general segmentation techniques (Zygomalas et al., 2016). In this paper, the new hybrid methods are discussed. Figure 2 illustrates the design of the proposed work. The research was carried out based on the concepts of research methodology and development using the software architecture pattern Model View Controller (MVC), which is used in most of the medical imaging research. The Model represents the volumetric data in R^3^ and the associated image processing methods (includes hybrid methods of our objectives). The View is the visualization of the model using either 2D MPR images or 3D volume rendering methods.

In addition, the Controller is the user who interacts with the model through the view. A CTC CAA prototype software has been developed with different features what is expected from a medical imaging application. The results were statistically validated.

*Research design*

According to the intent and the method of the study, this is an exploratory and experimental research type. The nature of this research seeks both qualitative and quantitative approaches. The stratified sampling was considered for sample design (desired n=150). Systemic bias and sampling error were minimized by selecting more number of samples randomly from the infinite population (N=824). Reliability, suitability and adequacy of CTC images were checked. The volumetric overlap computation (for segmentation) and parametric testing procedure ‘paired t test’ (for polyp measurement) was applied to validate the results. In this retrospective study, the anonymized clinical dataset was obtained from Cancer Imaging Archive, USA (Clark et al., 2013). The image acquisition parameters are shown in Table 1. Dataset with motion artifacts, metal artifacts, and incomplete colon distention were discarded. The CTC image completeness was checked for type 1 and type 2 attributes for the compatibility with the latest DICOM standard (DICOM PS3.3, 2012).

*Colon segmentation*

**Figure 3:**
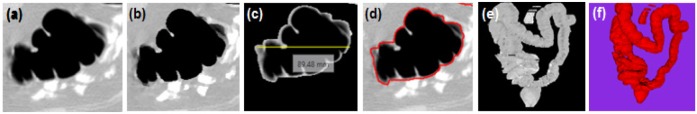
Results of Each Step: a, Original axial slice; b, Adaptive smoothing; c, Colonic content preserved after canny and CCL method; d, The boundary delineation; e, DRR of VOI, f) Surface rendered image of VOI

The analysis of polyps requires a segmented colon (Volume Of Interest - VOI) from the set of 2D images. A new boundary based hybrid method of colon segmentation is developed with the domain knowledge of colonic distention (Radiologist’s perspective of colon distention measurement) grading (Sliesenger et. al, 2010; Summers et. al, 2009). Eq. 1 gives the relation between the unsegmented colon and the VOI where S_o_ is the segmented colon (V_b_), S is the unsegmented colon (V_g_),c is the scene domain and f, f_0_ are the voxel intensities. The method is a multi-step approach which includes removal of noise from colon lumen without tampering the edges of polyps and soft tissue structures using adaptive smoothing filter (Figure 3b), the canny operator (Eq. 2-5) for recognizing colon boundary (3c) and Connected Component Labeling (CCL) method for delineating the colon boundary (3d). Canny operator results in the blob (large binary object) which is a collection of boundary points with value 1. Consider the *min* and *max* values of boundary points in both* x* and *y* axis. Then, within the colon boundary stitch the points between extreme left and right for each horizontal scan line and between the top and the bottom for each vertical scan line. This results in reaching the base of the colonic structures from where they are projected towards the colon lumen. For the pixels on the scan line, the HU intensities are copied from the original CT slice. This retains the colonic content without loss of any structures.

Lastly, the colonic segments were retained based on the diameter measured on axial slices (discarding blobs measuring<2cm, 3c). This forms the knowledge base in this approach. With this information of colon distention grading, only the colon (VOI) was retained and the other anatomical structures were discarded (3e).


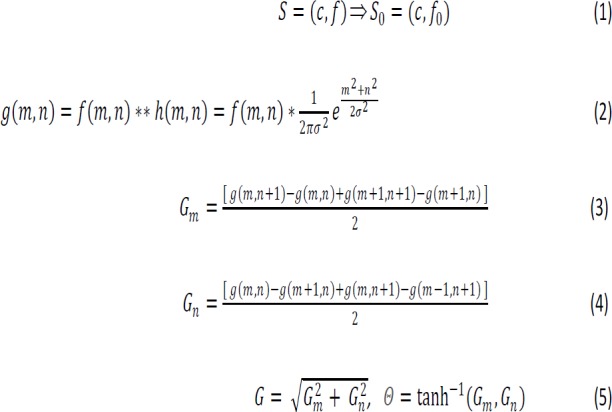


In these equations, h(m,n) is the Gaussian smoothing kernel, f(m,n) is the pixel intensity, ** is the convolution operator in *R*^2^, G is the gradient magnitude, G_m_ and G_n_ are the gradients, Θ is the gradient direction used in non-maximum suppression step to compare the edge strength of the current pixel with the pixels in the direction of Θ. For Gaussian smoothing, 5x5 filter kernel and standard deviation σ=1.4 are applied. The strong edges are tracked by Hysteresis thresholding. Here, to know whether the pixel is a candidate of edge, the pixels are compared with Low Threshold and High Threshold. It is suggested to use HT,LT in the range 2:1 to 3:1 respectively (Boyle et. al, 2008). The intensities in the range 40:100-50:120 (LT:HT) resulted in the best segmentation. The systematic results are shown in Figure 3.

*Electronic cleansing*

The tagged colonic materials from the colon are removed using an image post-processing technique called Electronic Cleansing (EC). The objective is to process the tagged fecal matter and other unwanted colonic materials from the CTC dataset acquired with various kVp values. An automated method has been developed with the domain knowledge of intensities of colonic contents at specific kVp. The range of attenuation coefficients is the key information to decide the abnormalities using the radiology images. The prior knowledge of Hounsfield Unit (HU) of colonic contents (air, fecal tagging agent, soft tissue, and fat) at specific kVp values is primarily considered in this approach. The theoretical knowledge of attenuation coefficients (NIST 2017; ICRU 2017) of colonic contents at various kVp is considered when the practical values of HU are not available for the study purpose. At 120kVp images, the HU of colonic contents are polyp: +54±5HU, lipoma (fat): -89±10HU, high density contrast: +130HU, air: -1,000±10HU and low attenuation gas: -900±25HU. In addition, this HU varies for different kVp images. The overall conversion from attenuation coefficients to display the pixel intensities is given by (Eq. 6).


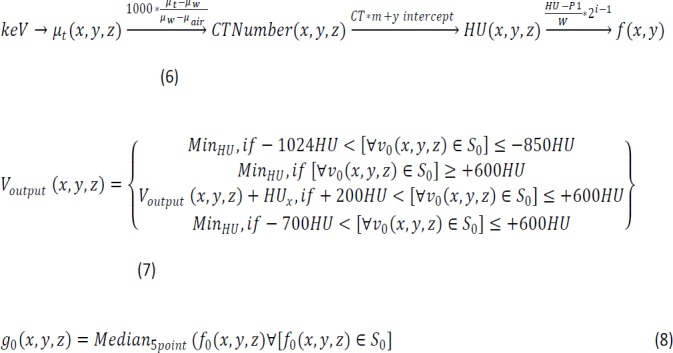


**Figure 4:**
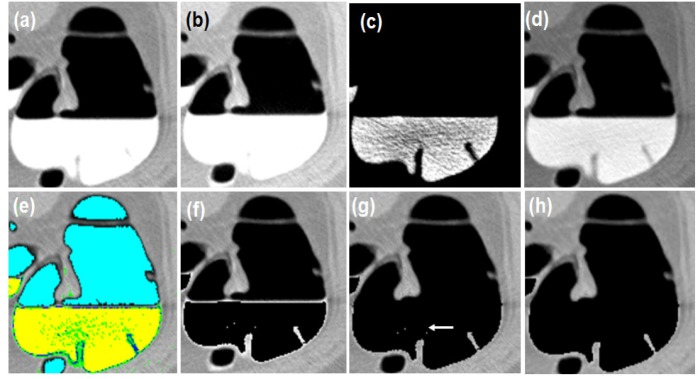
The Result of Each Step is Shown on Axial Images; (a-d) Unprocessed CT slices with different window center and window width. a, -200C, 1,500W; b, -350C, 14,00W; c, 0C, 2,000W; d, custom 600C, –400W; e, different areas with specific HU are shown in different colors; f, Pseudo enhanced intensities near the boundary of tissues; g, Air-contrast layer is removed and pseudo enhanced voxels are corrected; h, Noise removed

In these steps, μ_air,μ_w,μ_t are the attenuation coefficients of air, water, and tissue, respectively. HU is the tissue intensity computed w.r.t μ_w (μ_air is 0), W is the window value, C is the window center, and P1 is the window left border which are used to calculate the desired range of HU of different anatomies. i is the number of bits used to display the output gray intensities f(x,y) in the range [0..L-1]=[2^i-1^]. A lookup table of theoretically calculated HU is created (Eq. 6) and is used in the processing steps. The sequence of steps (Eq. 7) includes deducing the intensities of tagged materials, retaining the tissue structures using selective calculated HU from the look up table, removal of air-contrast boundary, and filtering (Eq. 8) the additive noise, which has resulted because of endoluminal fluids. The magic numbers are the practically observed HU at 120kVp. For demonstration purpose, the specific ranges of HU corresponding to colonic contents are shown in specific colors in Figure. 4e. The results of each step are shown on axial slice in Figure 4 (f-h).

**Figure 5:**
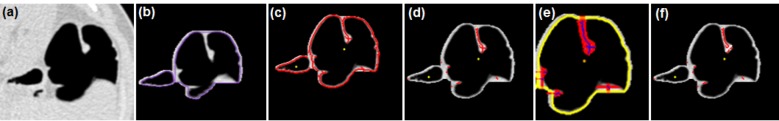
Step by Step Result of Polyp Measurement. a, Original axial slice showing a small polyp on Haustral fold; b, Boundary after colon segmentation; c, Skeletonization; d, Medial axis of the desired colonic structure; e, The structure reconstructed from the medial axis; f, The width of the polyp measured on Haustral fold

**Figure 6:**
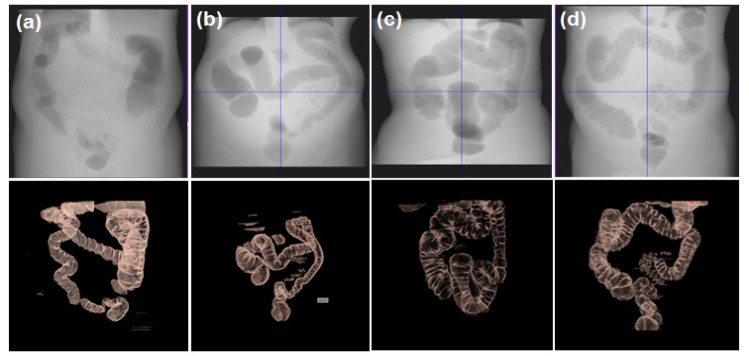
Colon before and after Segmentation; Row 1, DRR of the unsegmented colon; row 2, volume-rendered images of the segmented colon

**Figure. 7:**
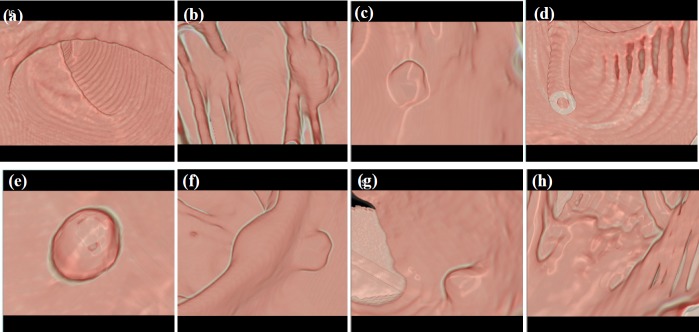
Endoluminal View of the Segmented Colon. a, Rectum; b, Tumor mass on Haustral fold; c, A sessile polyp; d, Air insufflation to colon through a tube; e, pedunculated polyp; f, A polyp on Haustral fold; g, A small polyp; h, Floating debris

**Figure 8:**
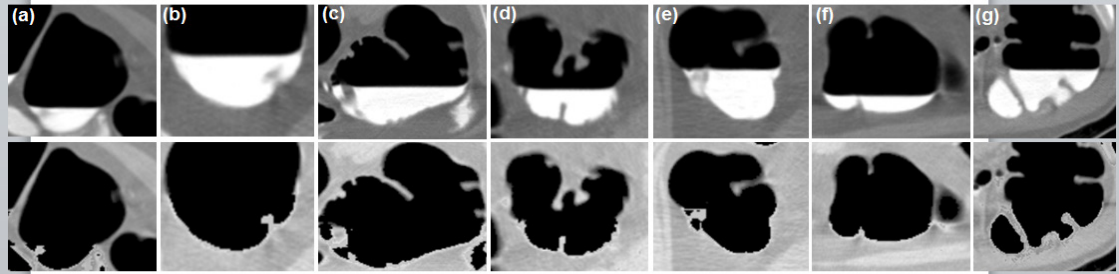
Unprocessed Axial Slices (First Row) and Virtually Cleansed Colon (Second Row)

**Figure 9:**
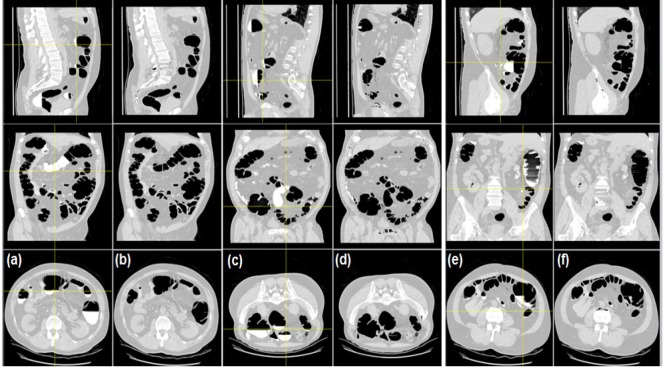
The MPR Planes of CT Volume with Left to Right (Axial), Anterior to Posterior and Axial Views (from the Top). Column a, b, c) Before cleansing and column a1, b1, c1) after cleaning of the colon. The colonic structures are preserved

**Figure 10:**
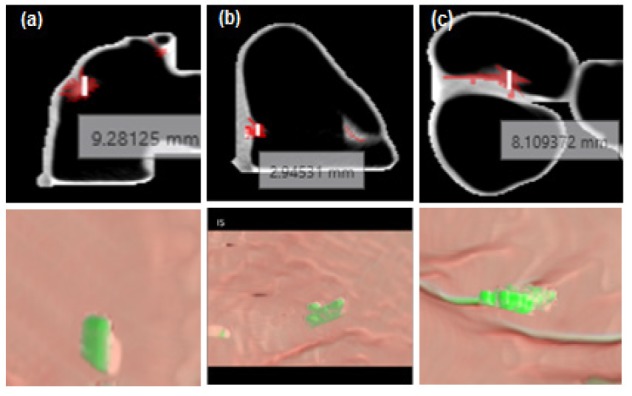
The CAA Result of the Smaller Polyp. Row 1 – measurements are shown on 2D axial MPR in mm and row 2– the Endoluminal view indicating the polyp in green color

**Figure 11:**
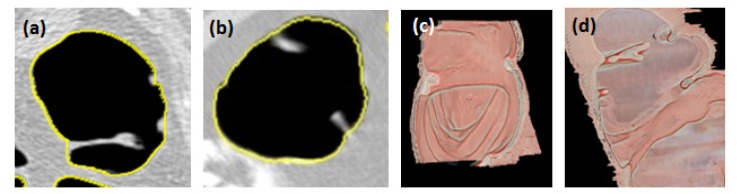
The Base of the Structures Identified. (a, b) Shown with yellow color on 2D MPR and (c, d) 3D view of a cross-section of the colonic interior

**Figure 12:**
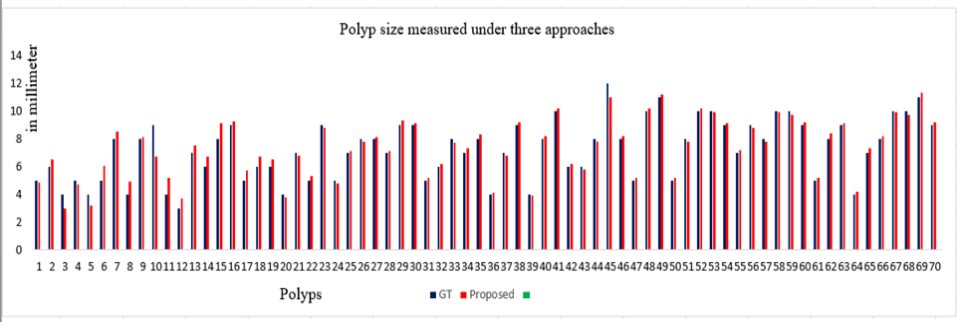
Graph of Polyp Measurement Under Two Approaches. Y-axis shows the measurement in mm

**Table 1:** Parameters of CTC Image Acquisition

Parameter	Value
Scanner	Siemens SOMATOMTM 16, 64 slices and GE LightSpeedTM 16 slices
ST	1.25mm, 2.5mm
kVp	100, 120
mA	200, 240, 250, 300
Pixel resolution	512, 512
Pixel size	{0.546875..0.9765625}mm
Scan positions	Feet First Supine (FFS) and Prone (FFP)
No. of Images	1000 CT images/patient
MDCT	4 and 8 slices
Patient age	{40-80}yrs.

*Measurement of polyp size*

Polyps are measured in CAA systems mainly using shape and size. The objective of this work is to measure accurately the smaller polyps, which help the Radiologist in making clinical decisions. A method with morphological image processing using domain knowledge of colon polyp analysis is developed. The basic requirement for polyp size measurement is locating the base of the colonic structures (Figure 11 a-d) which has been done in our colon segmentation method. In the next step, the morphological processing operator skeletonization is applied to identify the thin version (5c) of the segmented object (S_0_) (5b). Skeletonization gives the thin version of the shape irrespective of the congruency ( ≅ ) of the structure.

(9) $$\mathrm{Accuracy}=\frac{A\cap B}{A\cup B}\mathrm{*100}$$

The intersection of skeleton points and the colon boundary points of S_0_ is computed and then the result is negated. This retains the medial axis (S_k_^1^) of the colonic structures (Figure 5d). For each point on S_k_^1^ the orthogonal lines are computed using GramSchmidt orthogonalization and a line with maximum diameter is considered as the width of the shape. In the next step, based on the domain information the structure is categorized as polyp if its height is less than 6mm and a candidate of Haustral fold otherwise. Based on the height to width ratio, these small structures are categorized as sessile (if height is greater than 1.5 times its width) or flat polyps (if otherwise) (Summers et al., 2009). The result of each step is shown in Figure 5. Figure 5f shows a sessile polyp measured on Haustral fold whose width is shown using the white color line. 

## Results

In empirical testing, forty CTC dataset was considered. The results of each objective are shown in Figures. 6-10. The results were evaluated on both 2D and 3D views. The X-ray (DRR – Digitally Reconstructed Radiograph) image of the unsegmented and the volume rendered segmented colon are shown in Figure 6 (a-d) and 6e-h respectively. These DRR images in all three MPR directions help in qualitative assessment of the segmentation results. Post colon segmentation, different cases of colonic interiors are shown in Figure 7 with direct volume rendering method. The results of different clinical cases of EC are shown on axial slices in Figure. 8. It illustrates the partially and completely submerged structures in the contrast agent. Smaller polyps can be seen in Figures. 8a and 8e. Post EC, these structures are preserved without erosion and the pseudo enhanced voxel intensities are also corrected. The Haustral fold near the air-tagging boundary and submerged in contrast has been retained without any artifact (Figure 8d, 8g).The pseudo enhanced tissues near the boundary are also corrected. Figure 9 illustrates the entire colon visualized with 2D MPR before and after EC. Smaller polyps delineated and their measurements are shown in Figure 10. In 2D, the measured polyps and its measurement are displayed using red color and tooltip, respectively. In 3D view, they are displayed in green.

## Discussion

**Colon segmentation:** Measuring the height and width of the polyp relies on the location from where the polyp arises. The problem of identifying the colon boundary from where the colonic structures protrude has successfully been addressed which is the key finding in this approach (Figure 11). This helps in accurate measurement of polyp height. The method resulted in closed boundary (no segmentation leaks) with a thickness of one pixel when compared with the other methods (3-4 pixels). The colonic structures were preserved after colon segmentation. Qualitatively, the results were validated through supervised evaluation technique where the DRR image of the unsegmented and the segmented colon were compared manually (Figure 6a-d). Volumetric overlap was computed (Eq. 9) between the results and the manually segmented boundary (GT - Ground Truth) for quantitative analysis. The average accuracy of 95.826±0.6854% was achieved when compared with GT.

**Electronic cleansing:** When the CTC images, which are acquired with different kVp values, are not available, we can rely on the knowledge of HU calculation for processing such images. The novelty of the proposed method is, HU are adaptively computed for colonic contents based on kVp values and these calculated values are used in EC. Only the desired colonic contents HU are processed. With this adaptive method, the submerged structures at different contrast levels are preserved, accentuated voxels near the soft tissues are corrected, and air-contrast layer is removed without tampering the adjacent tissues. Medium and full dose colon prepared images are cleansed without any artifacts. The results of different cases of EC are shown on axial slices in Figures. 8 and 9. The method was tested on dataset acquired at 100kVp and 120 kVp. For qualitative analysis, supervised evaluation method was applied to know the accuracy of the result. For quantitative validation, the sizes of nearly 30 polyps were measured on 2D axial slice and compared with the GT (Johnson, 2008). The minor sub-millimeter variation observed in the results was acceptable. 

**Polyp size measurement:** With pre-processing, effective colon segmentation and the knowledge of polyp measurement from doctor’s perspective, our method was able to detect polyps of size less than 6-9 mm. To avoid such polyps turning into cancer at a later stage, we have considered to measure polyp of size less than 5 mm, and let the radiologist decide on this result whether to consider them as significant of not. Skeletonization has outperformed compared to other methods since it delineates medial axis correctly irrespective of the congruency of the colonic structure. The polyps measured are shown in graph in Figure 12. The variation in measurements was studied between the GT and the proposed technique. The significant differences were measured using paired t-test. There were seventy polyps identified (n=70 TP) in control group (GT values) and experimental group. In group 1 (GT), μ ®_1=7.2571, σ_1_ =2.1447, Standard Error of Mean (SEM)=0.2563. In group 2 (our method), μ_2_=7.3577, σ_2_=2.0881 and SEM=0.2496. The calculated t value is 1.6771 at degree of freedom DOF=69 and the two tailed p-value is 0.098. As p>0.0001, at α=5%, the differences under two approaches were clinically not significant. For qualitative validation, the voting policy was considered. Since the number of samples is limited, per polyp analysis scheme was applied. The test results were TP=54, FP=3, FN=6 and TN=14. The statistics values were TPR=90% (sensitivity), TNR=82.3% (specificity) and accuracy=88.31%.

The proposed techniques were implemented on HP workstation with Intel Xeon^®^ CPU E52620 2.0GHz, 48GB DDR3 RAM, and 64bit OS. Microsoft volume rendering SDK (Melancon et al., 2013) and Marching Cube methods (Bourke, 2014) were used for object-based volume rendering. The prototype takes 3 sec for reading DICOM CT, 1 sec for DICOM validation, 1.5-2 min to segment the colon, 5 min for electronic cleansing and ~4.5 min for measuring the polyp from the dataset having 450-500 CTC images.

An automated system of colon polyp measurement has been developed to improve the existing CTC solutions. A new hybrid method of colon segmentation, which identified the mucous membrane correctly from where the polyp arises, was presented. In addition, the colonic structures were preserved without any loss. An adaptive method of the EC of the colon that works with the images of various levels of kVp, was presented. Based on the knowledge of height to width ratio of smaller polyps, an automated method for detection of polyps was presented. The research results were translated to a CTC prototype by considering the expectations from medical imaging systems. The software and clinical validation is in progress. After this, the prototype can be employed as a low-cost diagnosis tool for polyp detection. The exact comparison of these techniques cannot be made with the existing solutions as the software, dataset, the experts who evaluated these results vary, and most of them are clinical studies. Instead, the results are proved through the statistical analysis.

## Funding Statement

We are greatful to Manipal Academy of Higher Education for supporting this work under Dr. TMA Pai Ph.D. Program.
